# Hypertension in patients with hereditary thrombotic thrombocytopenic purpura

**DOI:** 10.1002/jha2.29

**Published:** 2020-06-10

**Authors:** Azra Borogovac, Erika Tarasco, Johanna A. Kremer Hovinga, James N. George

**Affiliations:** ^1^ Hematology‐Oncology Section Department of Medicine College of Medicine University of Oklahoma Health Sciences Center Oklahoma City Oklahoma; ^2^ Department of Biostatistics & Epidemiology Hudson College of Public Health University of Oklahoma Health Sciences Center Oklahoma City Oklahoma; ^3^ Department of Hematology and Central Hematology Laboratory Inselspital Bern University Hospital University of Bern Bern Switzerland; ^4^ Department for BioMedical Research University of Bern Bern Switzerland

**Keywords:** hereditary thrombotic thrombocytopenic purpura, hypertension, pregnancy, stroke

Stroke is a critical problem for patients with hereditary thrombotic thrombocytopenic purpura (TTP), often occurring at a young age. In 2019, two large registries reported that stroke occurred in 25% of 73 patients [1] and 31% of 120 patients [2]. A subsequent systematic review of 76 published case reports describing 155 patients with hereditary TTP reported a similar incidence of stroke (26%); the median age when stroke occurred was 19 years; 22% of strokes occurred in children <9 years old [3]. Hypertension is an important risk factor for stroke. Therefore, we asked, is the prevalence of hypertension increased in patients with hereditary TTP? However, hypertension was not mentioned in these three reports on stroke [1, 2, 3] or in a recent comprehensive review of hereditary TTP [4]. Therefore, we searched for the mention of hypertension in published case reports of patients with hereditary TTP [3] and reviewed the database of the International Hereditary TTP Registry [2].

Case reports of patients with hereditary TTP were identified searching MEDLINE and PubMed from 2001 (the year when ADAMTS13 was first described) to March 20, 2020, using our previously published methods [3]. Only patients with confirmed hereditary TTP, defined by ADAMTS13 activity <10% and biallelic *ADAMTS13* mutations, were included. Each article was searched for a report of blood pressure, the occurrence of hypertension, or the use of antihypertensive medication. We believe that bias to report hypertension may be minimal because the principal goal of most of these case reports was to describe new *ADAMTS13* mutations [3]. The International Hereditary TTP Registry has been previously described [2]; it has enrolled 139 patients with confirmed hereditary TTP through April, 2020. Because all prescribed medications taken by the enrolled patients are recorded in the Registry database, we identified hypertension by the use of antihypertensive medication.

Our updated review of case reports identified 180 patients in 80 articles. Blood pressure, diagnosis of hypertension, or taking antihypertensive medication was mentioned in the reports of 48 (27%) patients (Table 1). Nineteen (40%) of these 48 patients had hypertension that was not associated with pregnancy; each of these 19 patients is described in Table S1. The age when hypertension was diagnosed was reported for 12 patients; their age was 1.6‐39 years (median age, 7 years). Two additional patients were ages 15 and 18 years old when they were reported; hypertension had been diagnosed previously but the age of diagnosis was not reported. Five patients were described as having chronic hypertension at ages 44‐70; the age of onset of hypertension was not reported. Blood pressures were reported for only three of the 19 patients. Antihypertensive treatment was described for only one patient. Transient cerebral ischemic attack (TIA)/stroke and/or kidney failure was reported for 15 (79%) of these 19 patients.

**TABLE 1:** Frequency of hypertension in patients with hereditary thrombotic thrombocytopenic purpura (TTP). Panel A: A systematic review of published case reports. Panel B: Analysis of patients enrolled in the International Hereditary TTP Registry

Panel A: Systematic review of published case reports		
Patients	Articles	Description
132	51	Blood pressure or hypertension not reported
48 (27%)	29	Blood pressure or hypertension reported^a^
Blood pressure or hypertension reported (29 articles)		
14 (29%)	5	Blood pressure reported as normal
15 (31%)	11	Hypertension reported, attributed to pregnancy
19 (40%)	13	Hypertension reported, not associated with pregnancy. Age of onset was reported for 12 patients: range, 1.6‐39 years; median, 8 years

Panel B: International Hereditary TTP Registry		
Patients	Hypertension	Description
139	40 (29%)^b^	Age: range, 8‐88 years; median, 43 years. Age of onset of hypertension was not included in the registry database.

a Blood pressure was often reported only as normal or only as hypertension being present, without actual measurement data or requirement for antihypertensive treatment. One article reported patients without hypertension and also patients with hypertension. The 19 patients with hypertension not associated with pregnancy are individually described in the Table S1.

b Hypertension was identified by the patient's report of taking prescribed antihypertensive medication.

Among the other 29 patients, blood pressure was reported as normal in 14 patients and hypertension occurred during pregnancy in 15 patients.

Among the 139 patients enrolled in the International Hereditary TTP Registry, 40 (29%) were taking antihypertensive medications. Their age was 8‐88 years (median, 43 years). The Registry did not record the age when antihypertensive medication was begun. Twenty‐five (63%) of these 40 patients had had a TIA/stroke and 34 (85%) patients were being managed with regular plasma prophylaxis. In our previous report of 120 Registry patients [2], the frequency of TIA/stroke (31%) and plasma prophylaxis (71%) was less, suggesting that patients with hypertension had more severe manifestations of hereditary TTP.

Our question (Is the prevalence of hypertension increased in patients with hereditary TTP?) was also suggested by the increased prevalence of hypertension among patients who have recovered from acquired TTP [5]. We analyzed 43 patients who were diagnosed with their initial episode of acquired TTP at a median age of 39 years. At the time of diagnosis, their prevalence of hypertension (19%), defined by prescribed antihypertensive medication, was not different from the U.S. population matched for age, race, and gender (16%). After a median follow‐up of 7 years, the prevalence of hypertension (40%) in these 43 patients was significantly greater than the U.S. population matched for age, race, and gender (24%, *P* = .011) [5]. The increased occurrence of hypertension following recovery from acquired TTP may be related to the minor kidney injury that occurs in patients with acquired TTP.

Our data suggest that the frequency of hypertension in patients with hereditary TTP may be increased, especially in children and could contribute to their high risk for stroke [3]. The presence of chronic hypertension among women may contribute to their high risk for complications of pregnancy [4]. Chronic hypertension increases the risk for multiple complications: preeclampsia, fetal growth restriction, placental abruption, and preterm birth [6].

However, our data are insufficient to answer our question. These data do suggest that blood pressure should be documented in all evaluations of patients with hereditary TTP, especially in children and pregnant women. Recognition and effective control of hypertension should become part of each routine evaluation. Appropriate management of hypertension in patients with hereditary TTP may help to prevent stroke and complications of pregnancy.

## CONFLICT OF INTEREST

The authors declare no conflict of interest.

## Supporting information

SUPPORTING INFORMATION
