# Supplementary material for: Hypertension in patients with hereditary thrombotic thrombocytopenic purpura
Source: EJHaem. 2020 Jun 10;1(1):342–3. doi: 10.1002/jha2.29 (PMC9175757; doi:10.1002/jha2.29)
Supplement: Supplementary file 1 — SUPPORTING INFORMATION [file JHA2-1-342-s001.docx]

**Supplement Table 1. Description of 19 individual patients who were reported to have hypertension not associated with pregnancy, 2003-2020**

| **Pt,**  **Ref** | **Age** | **Patient Description** |
| --- | --- | --- |
| 1^1^ | 9 | Pt 1. HTN dx age 9, CKD age 10, ESRD age 14, kidney transplant age 15, resume dialysis age 23. No BP or antihypertensive treatment reported |
| 2^1^ | 7 | Pt 2. HTN dx age 7. TIA ages 15, 16. No BP or antihypertensive treatment reported |
| 3^2^ | 2 | Age 2, presented with intracranial bleeding after head injury. BP 160/120. Had several episodes of renal failure with HTN. No antihypertensive treatment reported |
| 4^3^ | 3 | Age 3, hemiparesis, BP 130/92 (HTN for age), Hgb 5.6, Plt 10,000. Age 7, stroke; age 11, hematuria, proteinuria. No antihypertensive treatment reported |
| 5^4^ | 11 | Hyperbilirubinemia at birth, exchange transfusion. Age 11, stroke, HTN. No BP or antihypertensive treatment reported |
| 6^5^ | 7 | Family 1, Pt 3. Current age 15, HTN since age 7 requiring antihypertensive medicine, moderate renal failure. Hyperbilirubinemia at birth, exchange transfusion. No BP reported |
| 7^5^ | NR | Family 2, Table: Pt number 6, age 18. Date of onset of HTN not reported. Hyperbilirubinemia at birth, exchange transfusion. Age 8, AKI, transient dialysis. No BP or antihypertensive treatment reported |
| 8^5^ | NR | Family 2, Table: Pt number 7, age 15. Date of onset of HTN not reported. Hyperbilirubinemia at birth, exchange transfusion. No BP or antihypertensive treatment reported |
| 9^6^ | 1.6 | Age 19 mo, presented with systolic BP 115-125 (>95^th^ percentile for age), Hgb 6.5, Plt 5000, SCr 0.7 mg/dL (normal, 0.3-0.7). No antihypertensive treatment reported |
| 10^7^ | 4 | HTN dx age 4, hematuria, MAHA, thrombocytopenia. No BP or antihypertensive treatment reported |
| 11^8^ | 7 | HTN dx age 7, thrombocytopenia, anemia. Splenectomy age 13, died with 1^st^ pregnancy, GA 28. No BP or antihypertensive treatment reported |
| 12^9^ | 39 | Age 39 presented with HTN, oliguria, SCr 4.6, MAHA, and thrombocytopenia. No BP or antihypertensive treatment reported |
| 13^10^ | NR | Age 32, multiple strokes. Age 48, ESRD, kidney transplant. History of HTN. No BP or antihypertensive treatment reported |
| 14^11^ | NR | Pt 14 (age not reported), recurrent thrombocytopenia, impaired kidney function, HTN. No BP or antihypertensive treatment reported |
| 15^11^ | 12 | Age 12, HTN, hemolysis, thrombocytopenia, kidney failure, cerebral hemorrhage. No BP or antihypertensive treatment reported |
| 16^12^ | NR | 70 yo woman with stroke, thrombocytopenia. History of HTN. At approximately age 30, 3 pregnancies, each with a diagnosis of DIC and AKI requiring dialysis. No BP or antihypertensive treatment reported |
| 17^13^ | 21 | Age 21, accelerated HTN, exudative retinopathy, Hgb 9.0, plt 22,000, Cr. 2.8. Renal biopsy with FSGS, mild tubulointerstitial chronicity. No BP or antihypertensive treatment reported |
| 18^14^ | NR | Table Pt 15, age 44, history of stroke, HTN, CKD. No BP or antihypertensive treatment reported |
| 19^14^ | NR | Table Pt 16, age 48, history of stroke, HTN. No BP or antihypertensive treatment reported |

The Age column reports the patient’s age when hypertension was diagnosed. NR, age when hypertension was diagnosed was not reported; for patient number 18, no age was reported. The Patient Description column identifies the patient in the text or tables of the cited article. Abbreviations: Pt, patient number; Ref, citation; Age, patient’s age when hypertension documented; HTN, hypertension; BP, blood pressure; AKI, acute kidney injury; CKD, chronic kidney disease; ESRD, end-stage renal disease; TIA, transient (cerebral) ischemic attack; MAHA, microangiopathic hemolytic anemia; GA, gestational age; Hgb, hemoglobin; Plt, platelets; SCr, serum creatinine; DIC, disseminated intravascular coagulation; FSGS, focal segmental glomerulosclerosis.

**REFERENCES**

1. Veyradier A, Obert B, Haddad E, et al. Severe deficiency of the specific von Willebrand factor-cleaving protease (ADAMTS 13) activity in a subgroup of children with atypical hemolytic uremic syndrome. J Pediatr 2003;142:310-7.

2. Shibagaki Y, Matsumoto M, Kokame K, et al. Novel compound heterozygote mutations (H234Q/R1206X) of the ADAMTS13 gene in an adult patient with Upshaw-Schulman syndrome showing predominant episodes of repeated acute renal failure. Nephrol Dial Transplant 2006;21:1289-92.

3. Park HW, Oh D, Kim N, et al. Congenital thrombotic thrombocytopenic purpura associated with unilateral moyamoya disease. Pediatr Nephrol 2008;23:1555-8.

4. Fujimura Y, Matsumoto M, Kokame K, et al. Pregnancy-induced thrombocytopenia and TTP, and the risk of fetal death, in Upshaw-Schulman syndrome: a series of 15 pregnancies in 9 genotyped patients. Br J Haematol 2009;144:742-54.

5. Klukowska A, Niewiadomska E, Budde U, Oyen F, Schneppenheim R. Difficulties in diagnosing congenital thrombotic thrombocytopenic purpura. J Pediatr Hematol Oncol 2010;32:103-7.

6. Prestidge TD, Rurali E, Wadsworth L, Wu JK, Moore JC, Bresin E. Congenital thrombotic thrombocytopenic purpura (cTTP) with two novel mutations. Pediatr Blood Cancer 2012;59:1296-8.

7. Alsultan A, Jarrar M, Al-Harbi T, M ALB. Novel frameshift mutations in ADAMTS13 in two families with hereditary thrombotic thrombocytopenic purpura. Pediatr Blood Cancer 2013;60:1559-60.

8. Drews K, Seremak-Mrozikiewicz A, Sobieszczyk S, Barlik M. Inherited thrombotic thrombocytopenic purpura in pregnancy. Neuro Endocrinol Lett 2013;34:508-13.

9. Krabbe JG, Kemna EW, Strunk AL, et al. Adult-onset congenital thrombotic thrombocytopenic purpura caused by a novel compound heterozygous mutation of the ADAMTS13 gene. Int J Hematol 2015;102:477-81.

10. Fattah H, Kumar D, George JN, et al. Successful kidney transplantation in a patient with congenital thrombotic thrombocytopenic purpura (Upshaw-Schulman syndrome). Transfusion 2017;57:3058-62.

11. Hassenpflug WA, Obser T, Bode J, et al. Genetic and Functional Characterization of ADAMTS13 Variants in a Patient Cohort with Upshaw-Schulman Syndrome Investigated in Germany. Thromb Haemost 2018;118:709-22.

12. Tenison E, Asif A, Sheridan M. Congenital thrombotic thrombocytopenic purpura presenting in adulthood with recurrent cerebrovascular events. BMJ Case Rep 2019;12.

13. Yadav RK, Ariga KK, Subbiah A, et al. Novel Heterozygous Mutations of Congenital Thrombotic Thrombocytopenic Purpura: A Rare Case Report. Indian J Nephrol 2019;29:295-7.

14. Pikovsky O, Arafat M, Ovadia H, et al. Congenital thrombotic thrombocytopenic purpura in a large cohort of patients carrying a novel mutation in ADAMTS13 gene. Thromb Res 2020;185:167-70.
